# The Voluntary Hospitals Commission

**Published:** 1923-05

**Authors:** 


### VOLUNTARY HOSPITALS COMMISSION.

THE Voluntary Hospitals Commission have now dealt with all the applications for grants in respect of 1921, except for a limited number of areas in Scotland in which Local Voluntary Hospital Committees were late in being established. The total deficits reported to the Commission amounted to £295,170 for London, £425,727 for the rest of England and Wales, and £15,340 for Scotland. The total deficits reported for the whole of Great Britain amounted to £736,237, as compared with the estimate of £1,000,000 by Lord Cave's Committee on which the Government grant of £500,000 was based. The following grants have now been made on the basis of pound for pound of new money raised or in sight, except in a limited number of cases in which emergency grants have been made to hospitals which had exhausted their realisable assets and without assistance from the Commission would have been compelled to close beds:—

| | |
|---|---|
| London .. .. .. .. .. | £225,000 |
| England and Wales (excluding London) .. | 194,242 |
| Scotland .. .. .. .. .. | 5,741 |
| Total .. .. .. .. | £424,983 |

The London hospitals have received their full share of the grant, but a balance of £75,017 still remains for distribution in the rest of England, Wales and Scotland. This balance has been apportioned on the basis of Lord Cave's estimate as follows:—

| | |
|---|---|
| England and Wales .. .. | £45,758 |
| Scotland .. .. .. | 29,259 |

The Local Voluntary Hospital Committees in England and Wales and the Consultative Committee in Scotland are being asked to formulate proposals, subject to the Treasury conditions, for the distribution of these balances towards the liquidation of the deficits which accumulated prior to 1921.

The Duke of Connaught has become Vice-Patron of the Royal Northern Hospital.

